# Early surgery versus optimal current step-up practice for chronic pancreatitis (ESCAPE): design and rationale of a randomized trial

**DOI:** 10.1186/1471-230X-13-49

**Published:** 2013-03-18

**Authors:** Usama Ahmed Ali, Yama Issa, Marco J Bruno, Harry van Goor, Hjalmar van Santvoort, Olivier RC Busch, Cornelis HC Dejong, Vincent B Nieuwenhuijs, Casper H van Eijck, Hendrik M van Dullemen, Paul Fockens, Peter D Siersema, Dirk J Gouma, Jeanin E van Hooft, Yolande Keulemans, Jan W Poley, Robin Timmer, Marc G Besselink, Frank P Vleggaar, Oliver H Wilder-Smith, Hein G Gooszen, Marcel GW Dijkgraaf, Marja A Boermeester

**Affiliations:** 1 Department of Surgery, Academic Medical Center Amsterdam, PO 22660, 1100 DD, Amsterdam, the Netherlands; 2 Department of Surgery, University Medical Center Utrecht, HP G04.228, PO 85500, 3508 GA, Utrecht, the Netherlands; 3 Department of Gastroenterology, Erasmus Medical Center Rotterdam, PO 2040, 3000 CA, Rotterdam, the Netherlands; 4 Department of Surgery, Radboud University Nijmegen Medical Center, HP 630, PO 9101, 6500 HB, Nijmegen, the Netherlands; 5 Department of Surgery, Maastricht University Medical Center, PO 5800, 6202 AZ, Maastricht, the Netherlands; 6 Department of Surgery, Isala Ziekenhuis, PO 10500, 8000 GM, Zwolle Zwolle, the Netherlands; 7 Department of Surgery, University Medical Center Groningen, PO 30001, 9700 RB, Groningen, the Netherlands; 8 Department of Surgery, Erasmus Medical Center Rotterdam, PO 2040, 3000 CA, Rotterdam, the Netherlands; 9 Department of Gastroenterology, University Medical Center Groningen, PO 30001, 9700 RB, Groningen, the Netherlands; 10 Department of Gastroenterology, Academic Medical Center Amsterdam, PO 22660, 1100 DD, Amsterdam, the Netherlands; 11 Department of Gastroenterology, University Medical Center Utrecht, PO 85500, 3508 GA, Utrecht, the Netherlands; 12 Department of Gastroenterology, Maastricht University Medical Center, PO 5800, 6202 AZ, Maastricht, the Netherlands; 13 Department of Gastroenterology, Antonius Ziekenhuis, PO 2500, 3430 EM, Nieuwegein, the Netherlands; 14 Department of Anesthesia, Radboud University Nijmegen Medical Center, HP 630, PO 9101, 6500 HB, Nijmegen, the Netherlands; 15 Department of Evidence Based Surgery, Radboud University Nijmegen Medical Center, HP 630, PO 9101, 6500 HB, Nijmegen, the Netherlands; 16 Clinical Research Unit, Academic Medical Center Amsterdam, PO 22660, 1100 DD, Amsterdam, the Netherlands

**Keywords:** Chronic pancreatitis, Pain, Surgical management, Surgery, Endoscopic treatment, Endoscopy, ERCP, Opioid, Pancreaticojejunostomy, Frey procedure

## Abstract

**Background:**

In current practice, patients with chronic pancreatitis undergo surgical intervention in a late stage of the disease, when conservative treatment and endoscopic interventions have failed. Recent evidence suggests that surgical intervention early on in the disease benefits patients in terms of better pain control and preservation of pancreatic function. Therefore, we designed a randomized controlled trial to evaluate the benefits, risks and costs of early surgical intervention compared to the current stepwise practice for chronic pancreatitis.

**Methods/design:**

The ESCAPE trial is a randomized controlled, parallel, superiority multicenter trial. Patients with chronic pancreatitis, a dilated pancreatic duct (≥ 5 mm) and moderate pain and/or frequent flare-ups will be registered and followed monthly as potential candidates for the trial. When a registered patient meets the randomization criteria (i.e. need for opioid analgesics) the patient will be randomized to either early surgical intervention (group A) or optimal current step-up practice (group B). An expert panel of chronic pancreatitis specialists will oversee the assessment of eligibility and ensure that allocation to either treatment arm is possible. Patients in group A will undergo pancreaticojejunostomy or a Frey-procedure in case of an enlarged pancreatic head (≥ 4 cm). Patients in group B will undergo a step-up practice of optimal medical treatment, if needed followed by endoscopic interventions, and if needed followed by surgery, according to predefined criteria. Primary outcome is pain assessed with the Izbicki pain score during a follow-up of 18 months. Secondary outcomes include complications, mortality, total direct and indirect costs, quality of life, pancreatic insufficiency, alternative pain scales, length of hospital admission, number of interventions and pancreatitis flare-ups. For the sample size calculation we defined a minimal clinically relevant difference in the primary endpoint as a difference of at least 15 points on the Izbicki pain score during follow-up. To detect this difference a total of 88 patients will be randomized (alpha 0.05, power 90%, drop-out 10%).

**Discussion:**

The ESCAPE trial will investigate whether early surgery in chronic pancreatitis is beneficial in terms of pain relief, pancreatic function and quality of life, compared with current step-up practice.

**Trial registration:**

ISRCTN: ISRCTN45877994

## Background

In patients with chronic pancreatitis (CP), the most important clinical problem is the management of pain, which often occurs daily and is disabling [1,2]. CP is also associated with pancreatic insufficiency, both endocrine and exocrine [3,4], which develops in 50% and 80% of patients within 5 years, respectively. CP is related to several causative factors, most notably alcohol toxicity. Other factors such as genetics, anatomic abnormalities and autoimmunity also play a role [5].

In current practice, patients with CP are managed by a conservative step-up practice. The first step is medical management, ranging from pancreatic enzymes and mild analgesics to opioids. When medical management fails, the next step is usually endoscopic intervention. Surgical intervention is kept as an option of last resort when other treatments have failed and the severity of disease has increased substantially and pain becomes unmanageable [6,7]. This approach is based on the so called ‘burnout hypothesis’, assuming that CP is a ‘self-limiting’ disease in which pain will ultimately resolve spontaneously due to progressive parenchymal destruction of the pancreas [3]. Many reports have questioned the validity of this hypothesis, including the group that first introduced it [4,8]. The main argument against the burnout hypothesis is the long time it takes for pain to subside; e.g. after 5 years of the onset of the disease about 60% of the patients will still experience substantial pain, and that in many reports complete insufficiency (‘burnout’) of the gland does not guarantee pain relief [4,8].

As an alternative to the current conservative step-up practice, there is emerging evidence to suggest that surgery early in the clinical course of CP is beneficial in terms of pain control and pancreatic function. First, pathophysiological studies of pain in CP have shown that prolonged periods of pain are associated with peripheral and central nerve sensitization. Consequently, a self-perpetuating pain state develops, which is very difficult to reverse and manage [9]. Secondly, experimental and clinical studies suggest that early surgical intervention can mitigate disease progression. In an experimental model of early versus late surgical drainage for CP in piglets, we have previously demonstrated that early surgery resulted in less histological cell damage and better exocrine pancreatic function [10]. Clinically, two observational cohort studies have shown that surgical interventions, especially drainage procedures, have the potential to delay the progressive loss of pancreatic function in CP patients [11-13]. We recently also performed an observational study that supports the hypothesis that longstanding disease is associated with poor pain control after surgical intervention. In 266 consecutive patients undergoing an operation for CP we observed that surgery after 3 years of onset of symptoms was independently associated with worsened pain outcome and increased rates of endocrine pancreatic insufficiency. Additionally, preoperative use of opioids (indicative of severity of disease and time-delay to surgery) were significantly associated with bad outcome of surgery in terms of pain control [14].

Finally, in a small pilot randomized trial, 32 patients with early stage CP and dilated pancreatic duct were randomized between early surgical drainage and a conservative approach [13,15]. Substantial pain relief was observed in 16/17 (94%) patients in the surgical group compared to 2/15 (13%) patients in the conservative group. New onset endocrine and exocrine pancreatic insufficiency were respectively observed in 2/13 (15%) and 1/15 (7%) in the early surgical group compared to 10/12 (83%) and 11/14 (79%) in the conservative group [13].

Despite the evidence suggesting a benefit of early surgery, most patients with chronic pancreatitis are not managed by this approach in current practice. Therefore, the Dutch Pancreatitis Study Group designed a randomized controlled multicenter trial to evaluate the benefits, risks and costs of early surgical intervention: the Early Surgery versus Optimal Current Step-up Practice for Chronic Pancreatitis (ESCAPE) – trial.

## Methods

### Study design

The ESCAPE trial is an open label randomized controlled, parallel, superiority multicenter trial.

## Objectives

### Study hypothesis

We hypothesize that early surgery can relieve pain early in the disease course of chronic pancreatitis. This will, consequently, improve patients’ health status, reduce health demand and costs and increase quality of life and social functioning.

### Research questions

• **Primary study question:** does early surgery, as compared to the current optimal step-up practice, provide better pain control in patients with CP and a dilated pancreatic duct?

• **Secondary study question:** What is the impact of early surgery in these patients in terms of severe complications, mortality, quality of life, costs, endocrine and exocrine pancreatic insufficiency and other clinical relevant outcomes?

## Study population

Patients with CP meeting the inclusion criteria for registration will be asked to participate in the study (registration informed consent). Consenting patients will be followed monthly as potential candidates for randomization. When a registered patient meets the randomization criterion he/she will be enrolled in the study after explicit informed consent. An expert panel of CP specialists (gastroenterologists, surgeons and radiologists) will oversee the assessment of eligibility and will ensure that allocation to either treatment arm is possible prior to randomization. Patients will be recruited from 24 hospitals of the Dutch Pancreatitis Study Group.

### Inclusion criteria

• Age > 18 years

• Confirmed CP: according to the following criteria (adapted from the M-ANNHEIM diagnostic criteria [16]):

▪ typical clinical history of chronic pancreatitis (i.e. recurrent pancreatitis or abdominal pain), and:

▪ one or more of the following additional criteria for the diagnosis of CP:

1. Pancreatic calcifications on CT or MRI imaging.

2. Moderate or marked ductal lesions (according to the Cambridge classification) on endoscopic retrograde cholangiopancreatography (ERCP) or magnetic resonance cholangiopancreatography (MRCP) imaging.

3. Marked and persistent exocrine insufficiency (defined as: a. pancreatic steatorrhea clearly relieved by enzyme supplementation, and/or b. fecal elastase levels of ≤ 200 micro gram/gram).

• Dilated pancreatic duct: dilated pancreatic duct of ≥ 5 mm, with or without enlargement of the pancreatic head [17].

• Presence of moderate, non-debilitating pain. This will be defined as chronic or recurrent abdominal pain sufficiently relieved with non-opioid analgesics.

### Randomization criteria (after fulfilling inclusion criteria for registration)

Patients will be eligible for randomization if one of the following criteria is met:

• In patients with chronic abdominal pain related to chronic pancreatitis: a need to upgrade pain medication from non-opioids to opioid analgesics (opioids needed at least 3 days per week) and persistently needed for at least 2 weeks.

• In patients with recurrent flare-ups interspaced with pain-free intervals: flare-ups that have occurred at least 3 times during one year (i.e. 12 months), with each flare-up fulfilling the following criteria:

▪ duration of at least 7 consecutive days;

▪ necessitate opioid use during flare-up;

▪ impairment of daily activities.

Informed consent must also be acquired for randomization.

### Exclusion criteria

**Figure 1:**
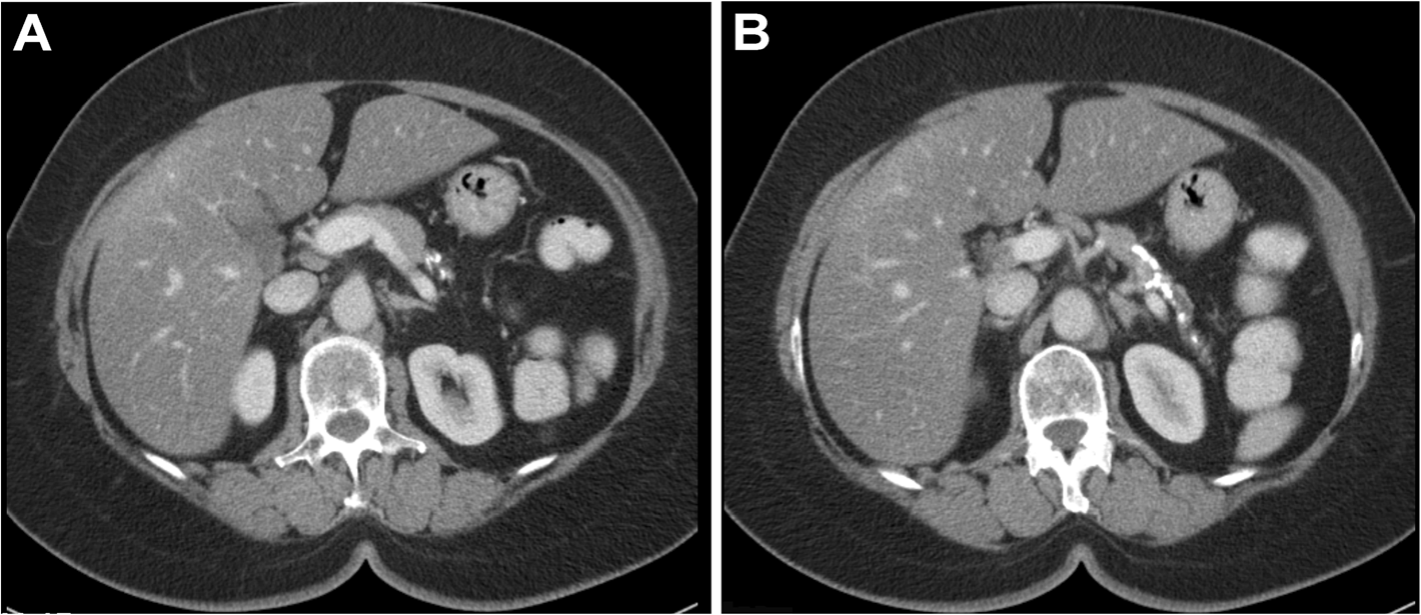
CT scan depicting stones and strictures exclusively located in the tail of the pancreas.

**Figure 2:**
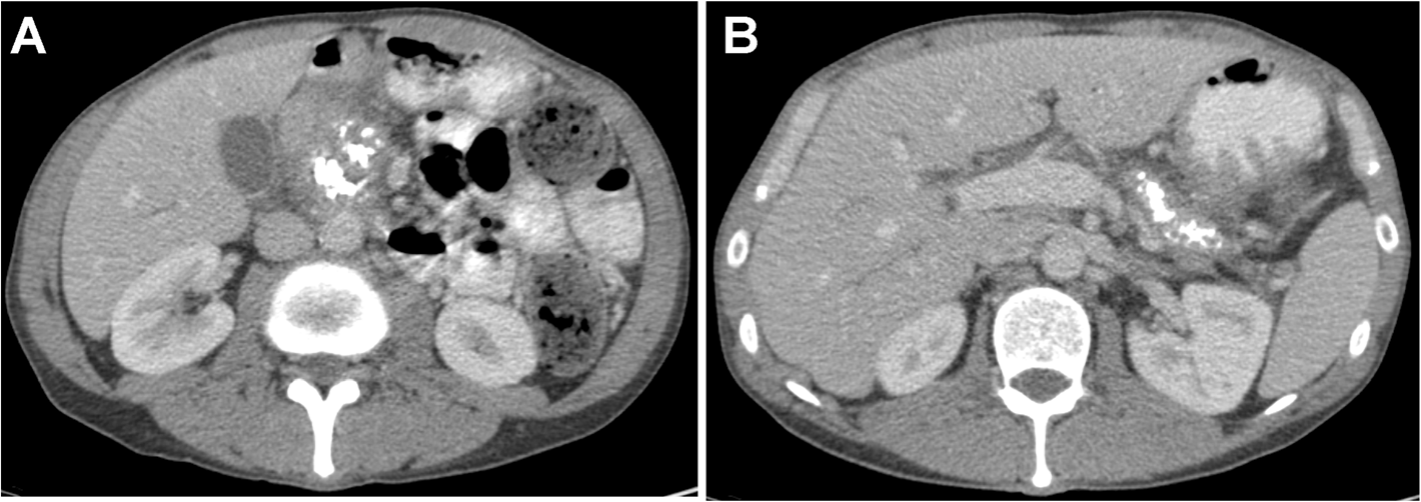
CT scan depicting fully impacted stones casting the entire main pancreatic duct from head (A) to tail (B).

• History of prolonged need of opioids: history for need for strong opioids for CP for a total period over 2 months or a history for need for weak opioids for CP for a total period of 6 months in the last 2 years. The following medications are considered weak opioids: codeine, dextropropoxyphene, dihydrocodeine, tramadol and partial agonists of morphine (e.g. pentazocine, butorphanol, nalbuphine).

• Previous pancreatic surgery

• Previous endoscopic dilatation or stenting of the pancreatic duct

• Episode of biliary obstruction in the previous 2 months (defined as jaundice or bilirubin levels ≥ 25 micromol / L) or the presence of a stent in the common bile duct

• Autoimmune pancreatitis (including elevated levels of gamma-globulins (IgG))

• Stones and strictures exclusively located in the tail of the pancreas (i.e. to the left of the vertebra) with relatively normal pancreatic head and corpus (Figure 1).

• Fully impacted stones casting the entire main pancreatic duct (from head to tail) and possibly side branches (Figure 2).

• Suspected or confirmed pancreatic malignancy

• Life expectancy of < 1 year for any reason

• Presence of duodenal obstruction necessitating surgery, as judged by the expert panel

• Presence of a pseudocyst larger than 6 cm necessitating intervention, as judged by the expert panel

• Contra-indications for surgery, as judged by the expert panel (e.g. American Society of Anesthesiology class IV, severe portal hypertension due to occluded portal vein)

• Pregnancy

## Study interventions

**Figure 3:**
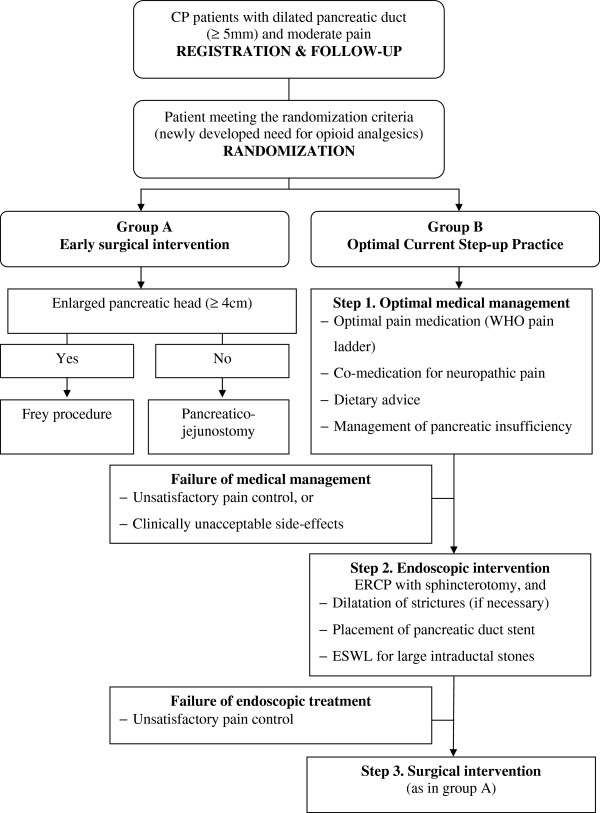
**Flow-diagram of study intervention arms in the ESCAPE-trial.** CP: chronic pancreatitis. ERCP: Endoscopic Retrograde Cholangiopancreaticography; ESWL: extracorporeal shock-wave lithotripsy. WHO: World Health Organization.

Patients will be randomly allocated to either early surgical intervention (group A) or step-up practice (group B). Figure 3 illustrates the management strategies in both intervention arms.

### Group A) early surgical intervention

The main aim of the surgical intervention will be surgical drainage of the dilated pancreatic duct. According to the size of the pancreatic head on the preoperative imaging (CT and MRI or ERCP), the following operation will be performed:

• In case of a non-enlarged pancreatic head (< 4 cm): surgical drainage of the pancreatic duct by a pancreaticojejunostomy.

• In case of an enlarged pancreatic head (≥ 4 cm): surgical drainage of the pancreatic duct and a duodenal-preserving pancreatic head resection (a Frey procedure).

Surgery will be performed within 6 weeks after randomization by an experienced pancreatic surgeon. Experience is defined as having previously performed at least 25 pancreatic operations for chronic pancreatitis, not including pancreatic cyst drainage.

#### Pancreaticojejunostomy

A pancreaticojejunostomy is performed as described by Partington and Rochelle [18]. The pancreatic duct is incised over its full length, from the tip of the tail to 2 cm from the ampulla. For removal of stones a V-shaped incision of the head may be necessary. The length of the anastomosis will be measured. Intra-operative photographs of the full length opened pancreatic duct will be taken to ensure that a uniform procedure was performed. If no pancreatic head enlargement is seen on preoperative imaging, but during the operation a markedly enlarged pancreatic head is found (≥ 4 cm), a Frey procedure is performed (see below).

#### Frey procedure

A duodenal-preserving resection of the pancreatic head, combined with a longitudinal pancreaticojejunostomy, is performed according to the technique described by Frey and Smith [19]. For the pancreaticojejunostomy, the pancreatic duct is incised over the full length (about 2 cm from the distal end of the tail to 2 cm from the ampulla. For reconstruction, one longitudinal pancreatojejunostomy is constructed, draining the resection cavity of the head and the full-length opened pancreatic duct. A biliodigestive anastomosis is performed in case of stenosis of the intrapancreatic segment of the common bile duct which cannot be decompressed by resecting the circumferential fibrous tissue.

### Group B) optimal current step-up practice

Patients randomized to the optimal current step-up practice will undergo the following stepwise treatment strategy as formulated by consensus of the Dutch Chronic Pancreatitis Expert Panel:

#### Step 1. Optimal medical management

Optimal pain control is attempted through the pain management guidelines as described below. A pain specialist and dietician will be consulted to optimize medical management. As long as pain control is achieved, the patient will remain in step 1.

Guidelines for this medical management are [20,21]:

Optimal pain medication according to the World Health Organization pain ladder, as follows:

○ Non-opioid medication: paracetamol (up to 4 daily doses of 1000 mg), or non-steroidal anti-inflammatory drugs (NSAIDs) (up to 3 daily doses of 75 mg of diclofenac or equivalent dose of other NSAIDs). When NSAIDs are used for a prolonged period of time, appropriate gastric protection should be given.

○ Weak opioids: tramadol (start: 3 daily doses of 50 mg used when needed; up to 4 daily doses of 100 mg).

○ Strong opioids: morphine, fentanyl, oxycodone, etc. Choice of medication and dose determined by treating physician (start morfine retard 2 daily doses of 10 mg or equivalent dose of another opioid; subsequent titration of dose should be guided by pain levels, development of tolerance and side-effects).

The type and dose of medication will be decided at the discretion of the treating physician and based on the clinical situation of the patient. As a guideline the physician should aim to maintain the pain VAS of the patient below a score of 4.

– Co-medication for neuropathic pain: in consultation with the pain specialist starting of one of the following co-medications for neuropathic pain is allowed.

○ Pregabaline (start: 2 daily doses of 75 mg; maximal dose: 2 daily doses of 300 mg).

○ Gabapentine (start: gradual increase over 3 days to a 3 daily dose of 300 mg; maximal dose: 3 daily doses of 1200 mg).

○ Amitryptyline (start: 25 mg per day before sleeping; maximal dose: 100 mg per day before sleeping).

– Dietary advice: patients will be referred for consultation with a dietician for analysis of nutritional status and starting of any necessary dietary program.

– Screening and management of pancreatic insufficiency at baseline:

○ Endocrine insufficiency will be screened by glucose level measurement. If endocrine insufficiency is established, the patient will be referred to a specialized internal medicine physician for management.

○ Exocrine insufficiency will be defined as presence of steatorrhoea, or fecal elastase levels at baseline of < 200 microgram/gram (measured in all patients as part of the study protocol – see ‘Outcome measures’). Patients with exocrine insufficiency will be treated with pancreatic enzyme substitution (start: equivalent to 25,000–50,000 FIP-E lipase with main meal, and 10,000–25,000 FIP-E lipase with in-between snacks; maximal dose: 10,000 FIP-E lipase/kg body weight per day).

#### Failure of medical management (failure of step 1)

If symptoms become intractable to medical management for a prolonged period of time despite adequate adjustment of medication, medical management is considered to have failed. This is defined as one of the following:

– Unsatisfactory pain control: defined as a VAS pain score > 4 in three follow-up moments (patients will be followed once every 2 weeks, see section ‘Data collection and follow-up’) despite adequate adjustment of medication.

– If achieving satisfactory pain control is associated with clinically unacceptable side-effects, the medication should be discontinued. Such side effects might include sedation, nausea, vomiting, constipation or itching.

#### Step 2. Endoscopic intervention

Patients with failed medical management will be referred for endoscopic intervention by an experienced endoscopist. Experience will be defined as having performed at least 50 therapeutic endoscopic interventions for chronic pancreatitis. The goal of the initial endoscopic intervention will be to drain the pancreatic duct by means of a pancreatic stent. At the end of endoscopic treatment of strictures and stones, as described below, adequate alleviation of pain should have been achieved.

1. *Stone removal:* in the absence of large (diameter ≥7 mm) intraductal stones in the pancreatic duct, patients will undergo an ERCP for stone removal. After sphincterotomy, a complete stone removal will be attempted. If stone removal is incomplete one or more pancreatic stents will be inserted and further stone removal will be attempted at the following ERCP. If large intraductal stones (diameter of ≥ 7 mm) are identified in the pancreatic duct on pre-intervention imaging studies, the patient will first undergo extracorporeal shock-wave lithotripsy (ESWL). After lithotripsy, stone fragments will be removed during the subsequent endoscopic intervention.

2. *Strictures and stent placement:* the decision for need of stricture dilation, with either a balloon catheter or a Soehendra catheter, will be made by the treating endoscopist. If strictures are present, a stent will be inserted in the pancreatic duct.

3. *Progressive stenting:* if a stent has been inserted, endoscopic treatment will be considered technically successful. From this point, the patient must have adequate pain control (with or without pain medication) in order to continue with endoscopic treatment (see section “Failure of endoscopic intervention”). Continuation of endoscopic treatment means the patients will undergo an elective endoscopic pancreatogram every 4 months. When complete runoff of contrast material is observed after removal of the stent and an extraction balloon can be passed through the pancreatic duct, endoscopic treatment is considered completed and stenting will be stopped. Persistent strictures will be treated by repeated endoscopic dilations and sequential insertion of new stents for a maximal period of 1 year, after which stenting must be stopped.

#### Failure of endoscopic intervention (failure of step 2)

Failure of endoscopic intervention is defined as:

Persistent pain after 12 weeks of endoscopic therapy (assessed from 8 weeks onwards after the start of endoscopic therapy): defined as a VAS pain score > 4 in at least three out of four follow-up moments (with or without pain medication). When no endoscopic treatment was found to be possible after attempting by a skilled endoscopist and pain persists, failure of endoscopic intervention will also be a fact.

#### Step 3. Surgical intervention

If endoscopic intervention fails, the third en last step will be surgical intervention. Surgical intervention will be done as described for group A.

## Outcome measures

### Primary outcome

The primary clinical outcome is the degree of pain as assessed by the Izbicki pain score at 2 weeks intervals during the follow-up period of 18 months. The Izbicki pain score is a validated pain score specifically designed for chronic pancreatitis [17,22]. It consists of four questions regarding frequency of pain, intensity of pain (VAS score), use of analgesics, and disease-related inability to work. Based on these question a pain score can be calculated ranging from 0 (no pain) to 100 (severe, debilitating pain).

### Secondary outcome

Secondary outcomes are:

• Severe complications related to disease progression, or endoscopic and surgical interventions (see Table 1 for definitions):

○ Mortality (all-cause)

○ Disease progression: development of pseudocysts, pancreatic insufficiency (endocrine or exocrine), duodenum obstruction, chronic use of opioids (defined as need for opioids for a period > 6 months), hospital admissions for CP up flares.

○ Endoscopic intervention: (acute) pancreatitis flare-up, cholangitis, acute cholecystitis, retroperitioneal or bowel perforation, haemorrhage.

○ Surgical intervention: anastomotic leakage, bleeding, sepsis, intra-abdominal abscesses, burst abdomen, pneumonia, severe wound infection (requiring prolonged hospital stay), severe delayed gastric emptying (requiring > 10 days of nasogastric intubation or inability to tolerate solid diet on or after the 14^th^ postoperative day), any relaparotomy for other reasons.

• Quality of life: assessed by the validated short form 36 (SF-36) and the EQ-5D questionnaires [23,24].

• Total direct and indirect costs

• Endocrine pancreatic insufficiency: determined by use of anti-diabetic medication or abnormal serum glucose levels (fasting serum glucose levels > 6.0 mmol/L in capillary blood, or > 6.9 mmol/L in venous plasma at two different days) [25].

• Exocrine pancreatic insufficiency: determined by fecal elastase levels (< 200 μg/g).

• Additional pain measurements: due to the heterogeneity in reporting of pain in previous trials, and in order for the results of this trial to be comparable with other studies, the following additional measures of pain will be reported as well:

○ Proportion of patients with complete and partial pain relief at end of follow-up, defined as follows [17]:

▪ Complete pain relief: an Izbicki pain score ≤ 10 points

▪ Partial pain relief: a decrease of >50% from baseline in the Izbicki score with a final score >10 points.

○ Visual analogue score (VAS) for pain: measured as part of the Izbicki score.

○ Büchler pain score: alternative pain measure based on the Izbicki questionnaire, and calculated by the multiplication of two of the four items of the Izbicki questionnaire (i.e. pain frequency and pain intensity) [26].

• Number and duration of hospital admissions during study period.

• Number of performed interventions: total number of endoscopic and surgical interventions, including initial intervention.

• Number of pancreatitis flare-ups during the study period.

**Table 1:** Definitions of complications

**Complications**	**Definition**
Pseudocysts	Fluid-filled collection in the pancreas without epithelial cover proven by CT.
Duodenum obstruction	Clinical symptoms suggestive of duodenal obstruction (retention, nausea, vomiting) and imaging evidence of duodenum obstruction by the pancreas.
Chronic use of opioids	Daily need for strong opioids for a period > 6 months.
(Acute) pancreatitis flare-up	Episode of upper abdominal pain requiring hospitalization with either increased amylase (>3 normal level) or typical upper abdominal pain recognized by patient from previous episodes.
Cholangitis	1) Body temperature > 38.5°C and 2) Bilirubin > 20 μmol/L and/ or common bile duct of > 8 mm for age ≤ 75 years or > 10 mm for age > 75 years on abdominal ultrasound or CT.
Acute cholecystitis	1) Local signs of inflammation (Murphy’s sign, right upper quadrant mass/pain/tenderness), 2) Systemic signs of inflammation (Fever, elevated CRP, elevated WBC) and 3) Gallstones on abdominal ultrasound.
Perforation	Retroperitoneal or bowel-wall perforation documented by any radiographic technique.
Anastomotic leakage: Pancreaticojejunostomy	High amylase level (>3 times serum amylase) in the abdominal drain fluid, or pancreatic leakage proven by imaging or at relaparotomy, often but not necessarily in combination with one or more clinical signs (abdominal pain, peritoneal tenderness, temperature above 38.5°C or WBC above 15 X 10^9^/l).
Anastomotic leakage: biliary leakage	Bilirubin in abdominal drain or dehiscence found at laparotomy, often but not necessarily in combination with one or more clinical signs (abdominal pain, peritoneal tenderness, temperature above 38.5°C or WBC above 15 x 10^9^/l).
Bleeding/ Hemorrhage	Any bleeding leading to relaparotomy or intervention.
Sepsis	Presence of two or more of the following: fever or hypothermia, leucocytosis or leucopenia, tachycardia, and tachypnea or a supernormal minute ventilation.
Intra-abdominal abscesses	Intra-abdominal fluid collection with positive cultures identified by ultrasonography or CT, associated with persistent fever and elevations of white blood cells.
Burst abdomen	Post-operative separation of the abdominal musculo-aponeurotic layers with protruding viscera.
Pneumonia	Combination of clinical signs (coughing, dyspnoea), with infiltrative abnormalities on chest X-ray, raised inflammatory parameters (WBC and CRP) and/or positive culture in sputum. In intubated patient a positive endotracheal culture is mandatory.
Severe wound infection	Infection occurring within 30 days after the operative procedure, and requiring hospitalization or intervention with subsequent prolonged hospital stay (otherwise considered as minor complication).
Severe delayed gastric emptying	Persistent need for nasogastric intubation of over 10 days or inability to tolerate solid diet on or after the 14^th^ postoperative day.

*CT*: computed tomography. *WBC*: white blood cell count. *CRP*: C-reactive protein.

## Randomization

Randomization will be performed using a central automated online assignment system ensuring random generation of allocation and concealment of allocation. Only after the expert panel ensures a patient fulfills the eligibility criteria, the patient’s information is entered into the central online randomization module, which subsequently allocates the patient to one of the two groups. Block randomisation with varying block size (2, 4 or 6) is used. The randomization will be stratified to take into account the presence of enlarged pancreatic head (not enlarged pancreatic head (< 4 cm) versus enlarged pancreatic head (≥ 4 cm)).

## Blinding

Due to the nature of the intervention, blinding of either patients or treating physicians is not feasible. To ensure reliability, all outcomes will be cross checked with data from primary sources by an independent investigator not involved in patient care and not involved in data analysis. In case of uncertainty about the definition of an outcome (e.g. complications), an independent adjudication committee will perform a blinded outcome assessment of that outcome variable in all patients.

## Follow-up and data collection

### Izbicki pain score

The Izbicki pain score will be assessed every two weeks during a follow-up period of 18 months. For this end, the Izbicki pain score will be assessed via a web questionnaire. Patients who do not have an email will be given a folder with Izbiki pain score forms and return envelops. Patients will be contacted by telephone every two weeks and reminded to fill in the questionnaire and send it to the trial coordinators. The Izbicki pain score is a one page questionnaire, easily completed in less than 3 minutes. The folder with the Izbicki score forms will be re-filled at every outpatient clinic visit (scheduled every 6 months).

### Data sets

A standardized case record form (CRF) will be used. A study nurse, not involved in patient care, will monitor the CRFs at all sites. Once a year a minimum of 10% of the CRF data, including the primary and secondary endpoints, will be verified with source data.

### Outpatient follow-up visits

Other outcomes will be collected during scheduled visits to the outpatient clinic at baseline and at intervals of 6 months for the duration of the study period. Standardized evaluation of symptoms and laboratory investigations, as outlined in the ‘Outcomes measures’ section, will be performed at each follow-up visit. Questionnaires will be mailed prior to follow-up visits and collected during the visits. The questionnaires will be processed by medical research staff that is unaware of the allocated treatment.

### Other data

Additionally, relevant data will be collected regarding any hospital admissions or interventions during the study period. Interventions encompass all surgical and endoscopic procedures (including initial surgery), placement of jejunal feeding tubes and nerve blocks.

## Sample size

The hypothesis of the study is that early surgery will result in an early and persistent reduction of pain complaints during the 18 months follow-up. Since pain is associated with many of the negative effects of CP on costs, social functioning and quality of life, the average reduction of pain during this period is of interest.

Calculation of a sample size based on the hypothesized difference between treatment arms was not possible due to unavailability of such data. We, therefore, decided to base this calculation on detecting the minimal clinically relevant difference of the Izbicki pain score. In a consensus meeting the Chronic Pancreatitis Expert Panel agreed that the trial should aim to detect a difference of 15 points or more on the Izbicki pain score (minimal clinically relevant difference) during follow-up. Attempting to detect smaller differences would not bring any clear advantage to the clinical decision making process, but would substantially increase the required sample size.

In previous studies, the average within group standard deviation of the Izbicki pain score was in the order of 20 points. Based on these assumptions, it was determined that 39 patients per group are needed to detect the minimal clinically relevant difference of 15 points on average using an ‘area under the curve’-analysis, based on alpha of 0.05 with a power of 90%. Taking into account a drop-out of 10%, a total sample size of 88 patients will randomized.

## Safety monitoring

An independent data and safety monitoring committee unaware of treatment assignment will evaluate the progress of the trial and will examine safety parameters at regular intervals (60 days after randomization of every 25 patients). All involved physicians will repetitively be asked to report any potential adverse events during the study period. These adverse events will be listed and discussed with the monitoring committee. The monitoring committee can ask for a full report in order to discuss a specific adverse event. The outcome of the meeting of the monitoring committee will be sent to the ethics board and the physicians involved. Any mortality will be directly reported to the safety committee and evaluated for cause of death and possible trial related serious adverse events. All serious adverse events will be reported online to the Central Committee on Research involving Human Subjects. Any death will also be reported to the central ethics board and to the local ethics board.

## Data and statistical analysis

**Figure 4:**
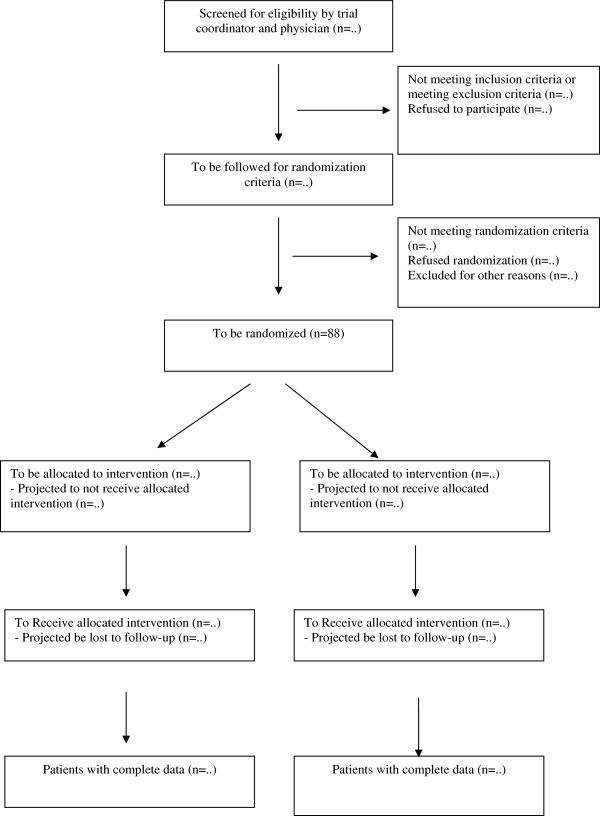
Pre-trial CONSORT chart of the ESCAPE trial.

Figure 4 depicts the pre-trial CONSORT diagram. The primary outcome of the study, the Izbicki pain scores with 2 weeks intervals during follow-up of 18 months, will be analyzed using an ‘area under the curve’ analysis. This allows for quantifying and comparing of the effect of study interventions on pain during the full follow-up period. Since we will measure the Izbicki pain score at fixed intervals, the ‘area under the curve’ analysis is statistically equivalent to comparing the average Izbicki score during the study period. For single missing values, linear interpolation will be used to impute this missing value. In case of lost-to-follow-up or drop-out, only the observed values will be used for analysis (no interpolation will be used). Secondary outcomes will be compared at the end of the follow-up. A predefined subgroup analysis in patients with chronic (continuous) pancreatitis pain and patients with recurrent pancreatitis flare-ups will be performed.

Analysis of primary and secondary outcomes will be conducted by a statistician blinded for the treatment allocation and not involved in data collection. Comparison of dichotomous outcomes will be performed using the Chi-squared test or Fisher’s exact test where appropriate. Results will be presented as number and percentages of events, as well as relative risk ratios with 95% confidence interval. For comparing continuous outcomes between two groups the Students t-test or the Mann-Whitney U test will be used, depending on the normality of the sample distribution as tested by the Kolmogorov-Smirnov test. A two-tailed P < 0.05 will be considered statistically significant. All analyses will be presented with 95% confidence intervals. If despite randomization baseline differences between the two groups were present, a sensitivity analysis using a multivariate regression approach will be performed correcting for any skewed baseline findings. Data will be analyzed on an intention-to-treat basis.

## Ethic and informed consent

The study will be conducted in accordance with the principles of the Declaration of Helsinki, and according to the laws governing human research in the Netherlands and Good Clinical Practice. The internal review board (IRB) of the Academic Medical Center Amsterdam approved the protocol. Secondary approval was obtained from the ethics committees of each participating hospital prior to commencement of randomization at the respective hospital. Informed consent will be obtained from each participating patient in oral and written form prior to randomization.

## Discussion

### Relevance of the study

The ESCAPE-trial is a multicenter trial designed to answer the question of whether early surgical intervention for CP will lead to better pain control and pancreatic function compared to the current step-up practice. Reviews and experts have repeatedly indicated a lack of randomized studies comparing surgical to medical treatment in CP [5,27]. This is widely considered as an important hiatus in the current knowledge regarding treatment of this disease. The ESCAPE-trial has, therefore, the potential to substantially improve the health status of CP patients and thereby impact daily clinical practice. The importance of the ESCAPE-trial is also strengthened by several reports indicating a substantial increase in incidence of CP in recent years [28,29].

### Rationale of study question

As described in the introduction, medical management is currently the first treatment for many CP patients. Although medical management may suffice to suppress symptoms early in the disease, it does little to influence the progression of disease and does not guarantee long-term relief of symptoms. Longitudinal studies show that of all CP patients, 40 to 75% will still require surgery for pain in the course of the disease [3,8,30,31]. The percentage of patients requiring a surgical intervention is probably even higher if only patients that are suitable candidates for surgery (have morphological abnormalities) are considered.

The current expectative management of CP means that, even in patients with clear morphological changes, progression of disease to the extent of severe and debilitating pain is a necessity before surgical interventions are considered. This approach should be questioned in the light of the prior discussed evidence (see introduction) suggesting that early intervention could mitigate disease progression and benefit the patient in terms of pain control as well as preservation of pancreatic function. The ESCAPE trial allows to examine these potential benefits in a setting of a well designed, sufficiently powered randomized trial.

### Rationale for used value of minimal clinically relevant difference

Calculation of a sample size based on the hypothesized difference between treatment arms was not possible, because the anticipated difference in the Izbicki pain scores between the early surgery group and the optimal current step-up practice could not be estimated with precision. Earlier surgical studies only provided Izbicki pain scores for patients who are operated late in the disease process. These scores do not reflect the expected results after early surgery, since we hypothesized that early surgery will achieve better pain control. In addition, the optimal current step-up practice is a consensus treatment designed for the purpose of this study. No exact levels of Izbicki scores are known for this research arm. The one small RCT by Nealon et al. that has studied the topic of early surgery in chronic pancreatitis only presented pain as proportion of patients with pain relief (94% for the early surgical group versus 13% for the non-surgical group) [13,15]. We choose not to use these values for our sample size calculations for several reasons:

– Measuring the outcome continuously best serves our hypothesis. It enables us to describe and better quantify the degree of pain during the 1.5 year follow-up period, and thus relate the degree of pain with costs and quality of life more precisely.

– The non-surgical arm in the above mentioned trial was not clearly described. In the ESCAPE-trial we optimized the management of those patients with our current practice step-approach.

– The observed differences in the trial are very large whereas its sample size is very small and no formal sample size calculation had been made; this needs confirmation in a large trial.

To be able to make a reliable sample size calculation we, therefore, decided to base this calculation on detecting the minimal clinically relevant difference in the average Izbicki pain scores between the two study groups. This is a recognized and well accepted method for sample size calculations [32,33]. First, we estimated the expected Izbicki pain score for the early surgery group. The most reliable Izbicki pain scores for the Dutch situation are produced by the CEPAN study which compared (late) surgery versus endoscopic intervention for CP [17]. The mean Izbicki pain score in the surgical group was 25 points. For the ESCAPE trial we hypothesize that an early surgical intervention will be able to achieve better pain control after surgery; probably in the range of 10 to 20 points. Subsequently, the clinical impact of an increase in the Izbicki pain score from this level by various amounts was discussed. A difference of 15 points was chosen to be the minimal difference that would alter the clinical situation of the patient in a potentially clinically relevant way. For illustration, the clinical meaning of such an increase is discussed: According to the Izbicki pain score, a patient with 15 points will have no pain to very mild pain (VAS 1 to 2), that occurs infrequently (several times a year), will not be in need for opioids medication and the disease will have minimal effect on the ability to work (no interfering to about 1 week per year of work disability). If the Izbicki pain score increases by 15 points (to 30 points) the situation of the patient will change slightly to the following: the patient will have mild pain (VAS 2.5 to 3.5), with a frequency of about once a month, necessitating weak opioids and interfering slightly with ability to work (about 1 week per year of work disability). Powering the trial to detect a smaller difference will increase the sample size substantially without affecting decision making, since smaller differences are likely not sufficient to change treatment choice from favouring step-up practice into favouring early surgery.

### Rationale for registration prior to randomization

In this trial, we aimed to select patients for which the two intervention arms are applicable in clinical practice. If patients can be managed satisfactory with non-opioid medication, there is no reason to seek more advanced treatment options. The low risks associated with non-opioid medication and adequate pain control do not justify a change of practice to more advanced modalities like early surgery. Progression of pain toward the need for opioid analgesics is a clear sign that pain management of a patient has become complex. We have, therefore, chosen this event as the most appropriate moment for randomization. Additionally, newly developed need for opioids is a transparent clinical event, thereby increasing the generalizability of the results to clinical practice.

### The expert panel

An Expert Panel of CP specialists (gastroenterologists, surgeons, pain specialists and radiologists) will oversee the assessment of eligibility and will ensure that allocation to either treatment arm is possible prior to randomization. The expert panel consists of 18 experts (8 surgeons, 7 gastroenterologists 2 anesthesiologists and 1 radiologists). The Expert Panel is already in use within the CARE project, a nationwide prospective registration and follow-up system for CP patients. Furthermore, the Expert Panel has already proven to be valuable and highly supportive during previous trial of the Dutch Pancreatitis Study Group [34-36].

### Formulation of the optimal current step-up practice arm

The current step-up practice arm represents optimal current practice in the management of chronic pancreatitis in the Netherlands. It is in concordance with international guidelines and current knowledge, and represents consensus of CP specialists within the Dutch Pancreatitis Study Group [6,7]. This optimal current step-up practice according to an explicit consensus protocol is considered the best alternative available for comparison to early surgery. An unstandardized control arm, leaving management decisions at the discretion of individual physicians is not preferred. From a clinical point of view it will be hard to know which treatment is compared in the trial. This will get in the way of drawing reliable conclusions and reduces the generalizability of the results.

Few published data exist about the proportion of patients that in current practice undergo surgery early in the disease. In a Dutch surgical cohort we observed that the median time between reported onset of first pain and endoscopic intervention was 16 months (10th - 90th percentile 2 to 56), while it took a median time of 15 months (10th-90th percentile 4 to 81) between first endoscopic treatment and surgical intervention [37]. In the same cohort patients underwent 0 to up to 29 endoscopic interventions before surgery [37]. In this trial endoscopic treatment is limited to 12 weeks if pain persists.

## Conclusion

The ESCAPE is a randomized controlled multicenter trial aiming to evaluate the benefits, risks and costs of early surgical intervention in patients with chronic pancreatitis as an alternative to current step-up practice of pain medication, if needed followed by endoscopic treatment, and eventually if needed followed by surgery.

## Abbreviations

CP: Chronic pancreatitis; CRF: Case record form; CT: Computed tomography; ERCP: Endoscopic retrograde cholangiopancreaticography; ESCAPE trial: Early surgery versus optimal current step-up practice for chronic pancreatitis trial; ESWL: Extracorporeal shock-wave lithotripsy; MRCP: Magnetic resonance cholangiopancreatography; NSAID: Non-steroidal anti-inflammatory drugs; SF-36: Short form 36; VAS: Visual analogue scale.

## Competing interests

The authors declare that they have no competing interests. MAB received unrestricted grants from Baxter, Abbott Laboratories, LifeCell, Ipsen for an unrelated projects. MJB received an unrestricted grant for unrelated projects from Axcan Pharma.

## Authors’ contributions

UAA drafted the manuscript. YI, HvS and MAB co-authored the writing of the manuscript. UAA, HGG, MGWD and MAB designed the study. All authors participated in the final design of the study during several meetings of the Dutch Pancreatitis Study Group. UAA and MGWD performed the sample size calculation. All authors edited the manuscript and read and approved the final manuscript.

## Authors’ information

The Dutch Pancreatitis Study Group (DPSG) was founded in 2002. The DPSG is a multi-disciplinary cooperation between 24 hospitals in the Netherlands (all 8 university medical centres in the country and 16 large teaching hospitals). Within this cooperation, all hospitals and specialties with expertise in clinical and experimental pancreatitis research are represented. Internist, gastroenterologist, surgeon and radiologists in these hospitals work together in this study group to advance the knowledge about and care for pancreatitis. The DPSG has an excellent track record in conducting multi-centre research and has in its 10 years of existence already conducted and/or initiated several large and demanding trials. The DPSG has already published results of three randomized controlled multicenter trials for the treatment of acute pancreatitis. Two other trials are currently ongoing.

These trials have concentrated on acute pancreatitis since this was the initial focus of the DPSG. Since 2008, the DPSG has extended its study scope to include CP. The ESCAPE trial will be, therefore, the first trial to be conducted for CP within this cooperation. Since the DPSG included all experts in the field of pancreatitis in the Netherlands, both acute and chronic, the ESCAPE trial will benefit from the effective and productive organizational infrastructure available.

## Pre-publication history

The pre-publication history for this paper can be accessed here:

http://www.biomedcentral.com/1471-230X/13/49/prepub
